# Exploring the Therapeutic Potential: Bioactive Molecules and Dietary Interventions in Multiple Sclerosis Management

**DOI:** 10.3390/cimb46060335

**Published:** 2024-06-03

**Authors:** Gabriele Tancreda, Silvia Ravera, Isabella Panfoli

**Affiliations:** 1 Department of Experimental Medicine, University of Genoa, 16132 Genoa, Italy; 2 Department of Pharmacy (DIFAR), University of Genoa, 16132 Genoa, Italy

**Keywords:** multiple sclerosis, neuroinflammation, neurodegeneration, oxidative stress, myelin, nutraceuticals, bioactive molecules, dietary interventions

## Abstract

Multiple sclerosis (MS) is a chronic autoimmune demyelinating disease of the central nervous system, the etiology of which is still unclear. Its hallmarks are inflammation and axonal damage. As a disease primarily impacting younger individuals, the social cost of MS is high. It has been proposed that environmental factors, smoking, and dietary habits acting on a genetic susceptibility play a role in MS. Recent studies indicate that diet can significantly influence the onset and progression of MS. This review delves into the impact of natural bioactive molecules on MS development and explores the dietary interventions that hold promise in managing the disease. Dietary patterns, including ketogenic and Mediterranean diets, are discussed. Theories about the potential mechanistic associations beneath the noted effects are also proposed. Several dietary components and patterns demonstrated the potential for a significant impact on MS. However, extensive prospective clinical trials are necessary to fully understand the role of natural bioactive molecules as disease modifiers in MS.

## 1. Multiple Sclerosis: An Overview

**Figure 1:**
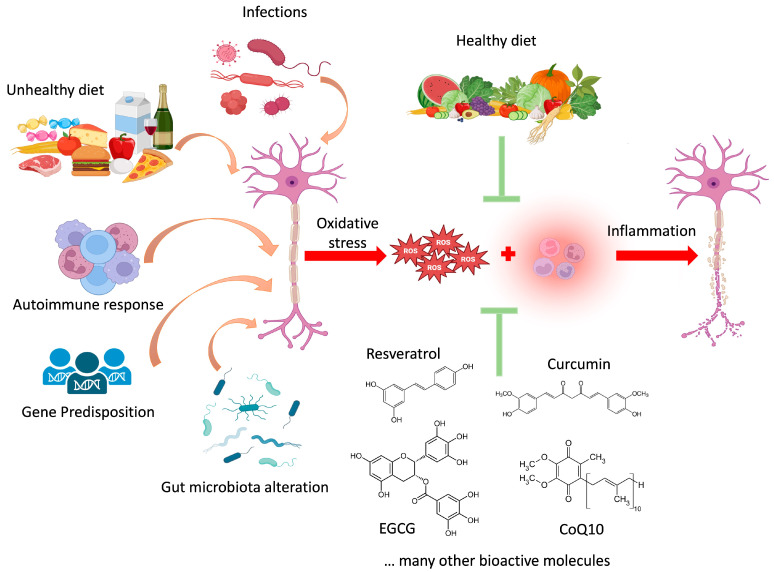
Schematic representation of the factors contributing (orange arrows) to oxidative stress and inflammation, the causes (red arrows) of axonal demyelination in MS, and the corrective factors (bioactive substances and a healthy diet) that could reduce (green arrows) the impact of the triggering factors. ROS: Reactive Oxygen Species; EGCG: Epigallocatechin gallate; CoQ10: Coenzyme Q10.

One of the main goals of nutritional and nutraceutical interventions in pathologies is to mitigate the leading and critical factors and their consequences, which are characteristic of the alteration itself. For this reason, nutritional therapies aim to restore the equal merits of biochemical and metabolic dysregulation, as well as the anatomical and structural impairments affected by the disease, as in the case of multiple sclerosis (MS) [1]. MS is the most common chronic inflammatory disease of the central nervous system (CNS) [2], the etiology of which is still unclear, although the associated demyelination is known to depend on an autoimmune reaction [2]. Among the various factors that could influence MS etiology, several environmental factors, such as low vitamin D, diet, and exposure to toxins, heavy metals, cigarette smoke, or viral (Epstein–Barr virus) molecular mimicry, seem to play a pivotal role (Figure 1) [3]. Interestingly, the role of environmental factors in the etiology of MS is supported by the fact that this disease is typical of industrialized countries, being most prevalent in North America (140 cases per 100,000), while the lowest prevalence is in sub-Saharan Africa (2.1 cases per 100,000) [4]. In addition, genome-wide association studies have linked a susceptibility to the major histocompatibility complex class *HLA-DRB1* gene [5]. MS is a chronic condition marked by physical disability and cognitive impairment, impacting quality of life [2]. In the early phase of MS (clinically isolated syndrome), inflammatory processes predominate, while degenerative processes are characteristic of late MS. The latter comprises relapsing-remitting MS (RRMS) and the development to secondary progressive MS (SPMS) in a decade. Less than 15% of patients display primary progressive MS (PPMS), characterized by rapidly progressing disability. Studies report physical and psychological symptoms ranging from depression to anxiety, mood changes, and cognitive mnemonic impairments [6]. The diagnosis of MS relies on demonstrating the presence of lesions in different CNS areas developing over time, identifiable using imaging, and laboratory data (finding of cerebral spinal fluid-specific oligoclonal bands), according to the Revised McDonald Criteria [7]. The disease-modifying therapies (DMTs) available for RRMS include interferons, glatiramer acetate, teriflunomide, sphingosine 1-phosphate receptor modulators, fumarates, cladribine, and three types of monoclonal antibodies [7]. The primary role of DMTs is to prevent relapses and future disability, although no DMT can suppress all relapses. There are two main treatment approaches for RRMS, i.e., starting with less potent medications and escalating to stronger ones if disease activity is observed, or initiating a medication with greater efficacy at the time of diagnosis, for better relapse control [2]. However, the latter approach exposes patients to a higher risk of adverse events [8].

Myelin is an abundant nervous system (NS) component produced by specialized glial cells, i.e., Schwann cells in the peripheral NS and oligodendrocytes in the CNS [9]. The white matter tracts consist of myelinated axons that account for ~40% of the human CNS volume [10]. Many axons are surrounded by myelin, a multilamellar membrane composed of proteins and lipids, such as myelin basic protein (MBP) and proteolipids, maintaining an essential role in sheath compaction and function. The myelin lipid component is composed of long-chain fatty acids, glycosphingolipids, and cholesterol [11]. The myelination process also requires several galactocerebrosides, the most abundant myelin glycolipids [12]. However, *D*-galactose (Gal) does not only seem to play a role in the lipid component of myelin, as it could also be used as an energy substrate for the CNS, causing a respiratory burst [13]. Moreover, Gal exerted a positive effect on MS-affected individuals in a previous clinical study [14,15].

Myelin has been long regarded as an inert insulating structure that allows rapid nerve signal transmission through saltatory conduction [11]. However, a consensus is rising on the trophic and metabolic supportive action of myelin [14,15]. An emerging function of myelin is the metabolic support of myelinated axons with small molecules [16]. As myelin sustains axonal integrity, the axon and its sheath should be considered a functional unit, which is structurally and metabolically coupled [8]. It was observed that the axonal energy metabolism is dependent on the associated glia [17]. The myelin is connected to the periaxonal space under the myelin sheath by cytoplasmic channels, allowing the movement of macromolecules [18]. The connexons may play a role in the radial transport of the macromolecules to the axoplasm, the blockage of which with oleamide slows down the axonal conduction velocity [19]. Long-term axonal survival requires trophic support from myelin [20]. As far as the macromolecules exchanged between neurons and glia are concerned, these have been alternatively supposed to be ATP [21,22] or monocarboxylates [23]. On the other hand, it was shown that the ATP demand rises when the axons spike at high frequencies [24]. Notably, the axonal mitochondria and myelin appear to be functionally related, as myelin lowers the need for the axonal mitochondria [25] and, conversely, the mitochondria increase in the sciatic nerve of a rat model of the demyelinating disease Charcot–Marie–Tooth disease type 1A [26]. Other authors have reported that demyelination increases the size of the axonal stationary mitochondria and the transport velocity of the motile ones [27], while remyelination reverts these effects. The incomplete insulation of the nerve fibers is linked to a dysfunctional propagation of the action potential, but also to axonal damage [11]. It appears that no myelin is better than defective myelin, as the loss of axon–glia metabolic coupling causes axonal transection and loss [20], as demonstrated in a post-mortem MS brain [28]. During SPMS, the chronically demyelinated axons degenerate due to the loss of glial trophic support [20,29], which is necessary for long-term axonal survival. Neurodegeneration contributes essentially to disability [30]. MS pathogenesis has been associated with oxidative stress. MS is a condition featuring neuroinflammation, an autoimmune process damaging the myelin and neurons due to high levels of oxidative stress. However, immunosuppression cannot revert the progression of the disease [31]. Overall, therefore, all these data suggest that dietary intervention may help modulate the certain cellular responses involved in inflammation and oxidative stress, directly support the trophic role of the sheath, or simply improve the psychophysical state of patients with MS. For this reason, in this review, we aimed to gather evidence from the scientific literature and clinical trials to highlight what could be the most useful, promising, and appropriate nutritional strategies, including the integration of nutraceutical and bioactive compounds, for the treatment of MS.

Therefore, since MS is often associated with severe inflammation due, in part, to impaired aerobic energy metabolism, this review focused on the bioactive molecules that exert a regulatory effect on inflammation and oxidative phosphorylation, basing the selection on the most recent literature data. The last systematic Cochrane review on dietary programs or supplementation for MS examined 30 studies on four main intervention types (dietary programs, intervention with polyunsaturated fatty acids, antioxidants, and other natural supplements) in subjects with RRMS. The evidence lacked a high certainty on whether any of these interventions could change the course of MS in patients but may have merit in improving their quality of life [32]. A systematic review was performed to evaluate the effects of dietary interventions on inflammatory markers in subjects with MS, searching electronic databases, hopefully the planned meta-analysis will establish the effect of diet on MS [33].

## 2. Natural Compounds of Interest in MS

### 2.1. Ubiquinone (CoQ10)

Coenzyme Q10 (CoQ10), also known as ubiquinone or ubiquinol, is an essential component of the electron transfer chain located in the mitochondrial inner membrane. The main competence of this hydrophilic vitamin-like molecule resides in the transport of electrons from complex I (NADH–ubiquinone reductase) or complex II (succinate–ubiquinone reductase) to complex III (cytochrome *c* reductase). The CoQ10 structure is at the basis of its function: it is composed of a benzoquinone ring, derived originally from the essential amino acid, phenylalanine, and a polyisoprene lipid trail that allows it to anchor itself into the mitochondrial membrane [34]. The ETC is ensured by the ability of CoQ10 to accept and donate electrons among the complex above, cited by switching from its fully reduced ubiquinol form to an oxidated one, ubiquinone. Ubiquinol possesses antioxidant properties and is responsible for the neutralization of free radicals and oxidative processes; thus, guaranteeing improved mitochondrial health [35]. Given its beneficial properties, various studies have investigated CoQ10 supplementation to improve the health parameters in different neurological conditions. A research group from the University of Medical Sciences in Theran explored how CoQ10 supplementation can ameliorate the inflammatory markers in patients with MS. The research group investigated markers such as matrix metalloproteinases (MMPs) and pro-inflammatory cytokines that can contribute to blood–brain barrier (BBB) permeability and lesion development in MS. A randomized double-blind placebo-controlled trial was carried out with 48 patients with MS (44 females, four males) with RRMS. The patients were divided into a placebo group (*n* = 24) and a CoQ10-supplemented group (100 mg five times per day, i.e., 500 mg/day, *n* = 24). The study lasted 12 weeks and aimed to assess the impact of CoQ10 supplementation on the inflammatory and anti-inflammatory markers in patients with MS and its potential as an adjunct therapy. The results from this study put forward evidence that CoQ10 supplementation led to significant reductions in the serum levels of Tumor Necrosis Factor Alfa (TNF-α), interleukin-6 (IL-6), and MMP-9 compared with placebo, while no significant changes were observed in the Transforming Growth Factor Beta and interleukin-4 (IL-4) levels. The CoQ10 ability to modulate MMP-9 levels emphasizes the relevance of this lipophilic compound not only in the prevention phase but also with the pathology in progress, since elevated MMP-9 levels have been found in acute MS lesions [36,37]. Another study attested CoQ10 as a potent free radical scavenger, a protector against lipid peroxidation, and an enhancer of antioxidant activity. This study involved 48 patients with RRMS who received either CoQ10 supplementation (500 mg/day) or placebo for 12 weeks. CoQ10 supplementation led to a reduction in malondialdehyde levels, a marker of lipid peroxidation, and an increase in the activity of antioxidant enzymes, particularly superoxide dismutase (SOD), even if no significant effect on glutathione peroxidase (GPx) activity or total antioxidant capacity was detected. However, the CoQ10 impact on clinical outcomes, such as the Expanded Disability Status Scale (EDSS), was not significant in this short-term study [38].

Fatigue and depression in MS are associated with inflammatory responses and oxidative stress [39]. Interestingly, CoQ10 has been suggested as a possible treatment due to its ability to decrease pro-inflammatory cytokines and protect brain cells. In this context, a research group recruited patients with MS and divided them into two groups: one receiving CoQ10 supplementation (500 mg/day) and the other receiving placebo. The effects of CoQ10 on fatigue and depression were assessed using standardized scales for fatigue severity and Beck’s Depression Inventory. The results showed that CoQ10 supplementation led to a significant decrease in both fatigue severity and depression scores compared with the placebo group. These improvements were attributed to CoQ10’s antioxidant and anti-inflammatory properties, which may help to counteract the inflammatory and neurodegenerative processes associated with MS-related fatigue and depression. The study suggests that CoQ10 supplementation could be a promising adjunctive therapy for managing fatigue and depression in patients with MS [40].

In addition, the effects of CoQ10 on remyelination, i.e., the restoring of the myelin sheath, in MS and inflammation have been investigated in a chronic MS model induced in the corpus callosum by the injection of cuprizone (CPZ), a copper chelator commonly used to induce demyelination in animal models. The study conducted by Behnam Khalilian and colleagues demonstrated that the CoQ10 administration can facilitate axonal remyelination. In detail, it was observed that in male mice treated with CPZ, CPZ plus sesame oil, or CPZ plus CoQ10, only the third treatment promoted remyelination, with an increase in the myelin content and the expression of myelin-related genes (MBP and Olig-1). Furthermore, CPZ poisoning caused a decrement in the antioxidant capacity and an increment in the oxidative stress in the brain tissue, which were reverted using CoQ10. Finally, CPZ increased the expression of pro-inflammatory cytokines (TNF-α and IL-6), which was attenuated using CoQ10 treatment [41]. The complexity of these studies highlights the efficacy and safety of CoQ10 integration at a dose of 500 mg/day for 12 weeks. These findings assessed the ameliorating effects of CoQ10 on the markers correlated with oxidative stress, levels of antioxidant enzymes, and depression. All the studies point out the need for further and larger investigation in clinical trials about the integration of CoQ10 for a longer period of administration.

### 2.2. Resveratrol

Resveratrol (RV), a natural polyphenol found in various plants, is considered one of the best bioactive molecules for MS management because it possesses several neuroprotective and anti-inflammatory properties, acting through multiple mechanisms [42,43,44]. Firstly, RV exerts a potent antioxidant effect, scavenging free radicals and reducing oxidative stress, which plays a crucial role in MS pathogenesis. In addition, RV enhances the expression and the activity of endogenous antioxidant enzymes, such as SOD, catalase, and GPx, through the activation of the nuclear factor erythroid 2-related factor 2 (Nrf2) signaling pathway [44,45,46]. In vitro experiments have demonstrated the protective effects of RV against oxidative stress-induced neuronal damage, including primary neuronal cultures and oligodendrocyte progenitor cells [47,48]. Moreover, RV displays anti-inflammatory properties, modulating several inflammatory pathways, inhibiting the production of pro-inflammatory cytokines, such as TNF-α, interleukin-1β (IL-1β), and IL-6, attenuating neuroinflammation, and reducing the immune-mediated damage to the CNS [49,50,51]. Additionally, RV promotes regulatory T cell (Treg) activation and suppresses pro-inflammatory T helper 17 cell activity, restoring the immune balance and dampening the autoimmune responses [52]. Preclinical studies utilizing animal models of MS, such as experimental autoimmune encephalomyelitis (EAE), have shown that RV supplementation attenuates oxidative stress markers, reduces neuroinflammation, and ameliorates the clinical symptoms of the disease [53].

RV also displays neuroprotective effects by enhancing neuronal survival and promoting remyelination as it stimulates the expression of neurotrophic factors, such as brain-derived neurotrophic factor and nerve growth factor, which support neuronal growth, differentiation, and repair [53]. In addition, RV enhances oligodendrocyte function and promotes the generation of myelin-producing cells, facilitating remyelination and restoring nerve conduction in demyelinated lesions [48].

### 2.3. Curcumin

Among the polyphenols, curcumin, the bioactive compound derived from the rhizome of *Curcuma longa*, exhibits immunomodulatory, antioxidant, neuroprotective, and remyelinating properties, which make it a good candidate as an adjuvant molecule in the treatment of MS [54]. In detail, curcumin exerts inhibitory effects on the autoimmune responses through interfering with the activation of nuclear factor kappa B, a key transcription factor involved in the regulation of the immune and inflammatory genes in MS [54]. The NF-κB inhibition causes a decrease in the release of pro-inflammatory cytokines, such as TNF-α, IL-1β, and IL-6 [55,56]. Curcumin also modulates the T-cell responses through regulating T-cell proliferation, differentiation, and cytokine production [57]. It inhibits the activation of T cells and promotes the generation of Tregs, which play a crucial role in maintaining immune tolerance and suppressing the autoimmune responses [58]. In addition, curcumin induces apoptosis in activated immune cells, such as T cells and macrophages, helping to restore immune homeostasis and prevent the development of further autoimmune diseases [59]. Regarding the oxidative stress associated with the autoimmune response in MS, curcumin enhances the antioxidant defense, acting as a scavenger of free radicals and increasing the activity of endogenous antioxidant enzymes, such as SOD and catalase, through the regulation of the KEAP1-Nrf2 pathway [60]. Moreover, curcumin slows down the oxidative stress production from oxidative phosphorylation metabolism by inhibiting FoF1 ATP synthase [61]. Besides the anti-inflammation and antioxidant effects, curcumin possesses neuroprotective properties, as it could promote neuronal survival and remyelination, as well as facilitate the axonal repair processes by modulating oligodendrocyte function, stimulating oligodendrocyte precursor cell (OPC) proliferation, and enhancing myelin production through the activation of the nuclear receptor PPAR-γ [62]. On the other hand, several clinical trials have investigated the therapeutic potential of curcumin in patients with MS [63,64].

### 2.4. Epigallocatechin Gallate (EGCG)

EGCG is a flavonoid polyphenolic compound belonging to the catechins sub-group, which is particularly present in green tea. This molecule has marked anti-inflammatory and antioxidant properties, and this makes it a compound of interest in various pathologies, including, in particular, autoimmune diseases such as MS [65]. EGCG could induce the repression of self-reactive T-cell proliferation, the reduction of pro-inflammatory cytokine production, and an increase in regulatory T-cell populations at the lymphoid tissue and CNS levels. Another important feature, especially in the MS context, is its ability to penetrate the BBB and accumulate within the neuron mitochondria, positively modulating oxidative stress [65,66]. The clinical trial in question is an experimental, prospective, mixed trial. The final sample of 51 patients was divided into two groups: one intervention and one placebo control, provided in capsules to be ingested daily. The intervention group was given a maintenance diet for 4 months, adapted to the preferences of the individual and divided into five daily meals, enriched with 60 mL of extra virgin coconut oil divided into two daily intakes of 30 mL each for breakfast and lunch, plus supplementation with 800 mg of EGCG provided by two capsules each of 400 mg with intake in the morning and afternoon. The control group followed the same isocaloric maintenance diet for 4 months as the intervention group, but they were given placebo in the form of capsules containing microcrystalline cellulose. The nutritional plan prescribed to both groups was structured following the pattern of the Mediterranean diet, with a subdivision of the three macronutrients compared with the total daily calorie intake as follows: 20% protein, 40% carbohydrates, and 40% Mediterranean lipids. The protein component of the diet observed the intake of proteins with a high biological value, derived from sources such as fresh fish, eggs, dairy products (milk, yogurt, and cheese), and vegetable proteins from cereals and legumes, at the expense of meat and its derivatives. The glucidic component was represented by complex carbohydrates, rich in fibers provided by rice, cereals, whole wheat bread, legumes, tubers, fruits, and vegetables. As for the lipids, the intake was mainly derived from extra virgin olive oil and nuts rich in omega 3 and omega 6, with a predominant intake of monounsaturated fatty acids with the intent to minimize the intake of saturated fatty acids. In the context of the analysis of the results deriving from this diet, it is important to highlight the high content of antioxidant and polyphenolic molecules; the intake related to this diet is estimated to be around 758.85 mg/kg of fresh food ingested. After 4 months of nutritional therapy, the intervention group showed a marked decrease in the serum concentration of IL-6, with also a concomitant improvement in the state of anxiety, and the functional capabilities of patients. Notably, the control group also reported a decrease in IL-6 levels; however, without improvements in the degree of anxiety or functional ability. Also noteworthy is a significant reduction in the body mass index (BMI) in both groups. The decrease in the IL-6 levels in both groups could be a consequence of the Mediterranean diet protocol followed in both cases [67]. The Mediterranean diet is characterized by a high intake of polyphenol molecules and antioxidants of various kinds, including vitamin C, vitamin E, carotenoids, and other polymeric molecules, which are responsible for safeguarding and protecting the organism at different levels [68]. Also, in both groups, a decrease in BMI was observed, which positively correlated with the plasma IL-6 levels.

The most marked differences highlighted by this study concerned the psychological quality of life parameters in patients. The association of EGCG and ketone bodies caused a significant improvement in the state of anxiety, also described as the immediate emotional state of response to an event or situation [67]. This can be explained thanks to the action of the EGCG, which is competent to increase the degree of inhibition in the CNS. In fact, EGCC interacts with the neurotransmitter, gamma-aminobutyric acid, which regulates neuronal excitability by reducing emotional tension with a sedative effect. Synergically with the effect just listed, ketone bodies are responsible for the amelioration of anxiety by inhibiting the activation of *N*-methyl-*D*-aspartate ionotropic receptors for glutamate, exerting stimulatory effects in the CNS [69]. Despite the limitations linked to the impossibility of attributing only the results of the study to coconut oil and EGCG, these two substances within a Mediterranean diet regimen have proven effective in significantly decreasing the state of anxiety and consequently improving cognitive functioning in patients with MS. The reduction of this biomarker might be due to the high intake of antioxidant and anti-inflammatory substances present in the Mediterranean dietary style.

### 2.5. Citicoline (DCI)

With regard to the remyelination process, a largely studied bioactive molecule is CDP-choline or DCI and its deriving metabolite, choline. DCI is the activated form of choline, identical to the natural precursor of phosphatidylcholine (PC) [70]. Choline plays a crucial role in remyelination through favoring the synthesis of numerous architectural elements of neurons’ membranes, such as phospholipids and sphingomyelin, another important constituent of myelin. The major phospholipid present in cell membranes is PC. In addition to the structural contribution, choline can be converted into acetylcholine, a neurotransmitter modulating synaptic transmission and promoting neuronal survival. Choline is a precursor for the synthesis of the methyl groups responsible for the epigenetic regulation of gene expression, which may enhance the differentiation and function of oligodendrocytes during remyelination [71]. Concerning DCI, optimal doses to promote remyelination in patients with MS are suggested to range from 500 mg to 2000 mg per day. Besides the previous beneficial effects cited for choline, DCI is reported to exert the modulation of the inflammatory response and oxidative stress, together with the promotion of neurogenesis. DCI has been shown to enhance the proliferation and differentiation of OPCs, which are crucial for remyelination in MS lesions. Significant results from a preclinical study of CPZ-induced demyelination in mice assessed a marked remyelination and oligodendrocyte differentiation 0,5 weeks after the suspension of the toxin from the diet. The activation of oligodendrocytes might also promote the reactivation of the remissive phase of RRMS. Considering the role and present use of this natural constituent in neurodegenerative disorders, such as Alzheimer’s disease and glaucoma, DCI should be highly considered as an adjunct in therapies for MS treatment in the prevention and ongoing phase [72].

### 2.6. Ellagic Acid

Ellagic acid is a phenolic compound derived from the hydrolysis of ellagitannins, which can be found in grapes, nuts, strawberries, black currents, raspberries, green tea, and pomegranates. It has been largely studied for its positive role in various pathologies characterized by oxidative stress [73]. In a preclinical trial, ellagic acid was dissolved and administered in drinking water at an estimated dose of 10 mg/kg/day 2 days before immunization and for the consequent 18 days to EAE female rats. Ellagic acid delayed the onset of neurological symptoms, positively impacted the progression of the disease, and prevented body weight loss compared with the placebo group. Significantly, ellagic acid preserved the content of MBP and sphingolipids in the cerebral cortex, resulting in the control non-immunized group favoring a protective mechanism against demyelination without affecting the onset of clinical signs [74]. In addition, ellagic acid appeared helpful in improving the depression in MS due to the alteration of the *L*-tyrosine pathway. In detail, the results of a randomized triple-blind placebo-controlled trial conducted in patients with mild-to-moderate depression showed that, after 12 weeks, the administration of 180 mg of ellagic acid (90 mg/day two times per day) caused a significant indoleamine 2,3-dioxygenase gene expression reduction that promoted *L*-tryptophane and serotonin level restoration. Moreover, the same treatment decreased interferon ƴ, nitric oxide, and cortisol concentrations, also exerting anti-inflammation and antioxidant effects.

### 2.7. Boswellic Acid (BA)

BA is another promising natural compound in MS treatment. This phytochemical represented in *Boswellia serrata* has gained attention for its anti-inflammatory, antioxidant, and neuroprotective properties [75]. Its principal mechanism of action includes the targeting of the nuclear Nrf2/heme oxygenase-1 (HO-1) signaling pathway, which is involved in the stimulation of the antioxidant and anti-inflammatory defenses. The enhancement of the Nrf2/HO-1 pathway induced by BA in experimental models of MS led to significant ameliorating effects related to neuroinflammation and neurological function, while also promoting myelin integrity [76]. The role of BA in neuroinflammation in MS is also due to its ability to suppress the production of pro-inflammatory cytokines, such as TNF-α, IL-1β, and IL-6, in immune cells and CNS-resident cells, thus reducing immune cell activation [77]. Moreover, BA interferes with the activation of the Nf-kB signaling pathway implicated in the transcription of pro-inflammatory cytokines, chemokines, and adhesion molecules, explaining the importance of this compound as an immune regulator in MS. By inhibiting the production of chemokines and leukocyte adhesion to endothelial cells, BA attenuates the phenomena of immune cell infiltration in the CNS, favoring BBB integrity and reducing neuroinflammation [78]. Relevant to mention in MS is the ability of BA to modulate MMP-9, an enzyme involved in the breakdown of extracellular matrix proteins, including BBB components [79]. BA is competent to modulate MMP-9 at various levels, the first being the direct inhibition of the genic expression and activity of the enzyme itself. Notably, BA is also responsible for suppressing the release of pro-inflammatory cytokines and chemokines implicated in MPP-9 regulation. Lastly, by exerting the potent antioxidant properties and scavenging free radicals, BA positively modulates oxidative stress, indirectly inhibiting MPP-9 activation. Despite being a promising candidate for MS treatment, clinical trials on BA are limited. So far, preliminary clinical trials and observational studies suggest that BA supplementation may improve clinical outcomes, reduce relapse rates, and alleviate MS symptoms, including fatigue, pain, and cognitive impairment [78].

### 2.8. Withania Somnifera Extract

In the context of molecules that are able to pass through the BBB, directly interact with the CNS, and modulate a plethora of metabolic pathways, phytochemicals from *Withania somnifera*, also known as Ashwagandha, might be interesting natural compounds to consider [80]. Ashwagandha belongs to the botanic species of *Solanaceae*, with a large history of use in the Ayurveda medicinal system [81]. It possesses a diverse phytochemical profile: (i) withanolides and naturally occurring steroidal lactones, which are considered the principal bioactive constituents responsible for the pharmacological properties ranging from anti-inflammatory to antioxidant, immunomodulatory, and neuroprotective. Notable withanolides found in Ashwagandha include withaferin A, withanolide A, and withanolide D; (ii) alkaloids: nitrogen-containing compounds, such as somniferine, somnine, and somniferinine, which contribute to the sedative, anxiolytic, and muscle relaxant effects of Ashwagandha; (iii) flavonoids that exert protection against oxidative stress in the brain. The most relevant in this Indian medical herb are kaempferol, quercetin, and rutin; (iv) tannins: polyphenolic compounds competent of antioxidant and anti-inflammatory properties as well; (v) saponins, such as glycosides, sitoindosides, and withanosides, which have been studied for their neuroprotective and adaptogenic properties. Furthermore, this molecule category favors the antioxidant and immune regulatory phenomena [82]. The neuroprotective mechanisms of Ashwagandha are the result of the synergy of these bioactive activities. Ashwagandha is capable of modulating antioxidant enzymes, suppressing neuroinflammation by inhibiting pro-inflammatory cytokines, reducing microglial activation, and, consequently, protecting neurons from damage and preserving the brain’s function. Besides, Ashwagandha promotes neurogenesis and neuronal regeneration by enhancing the proliferation and differentiation of neural stem cells. This process contributes to the repair and restoration of the neuronal circuits damaged in neurodegenerative conditions [82]. Clinical trials evidence the anxiolytic and antidepressant activity of this heterogeneous composed phytotherapy [83]. In a double-blind placebo-controlled clinical trial, 60 patients were enrolled and *n* = 30 were treated with 240 mg/day of Ashwagandha dry extract (standardized in not less than 84 mg of withanolide glycoside) and compared versus *n* = 30 receiving placebo. The study investigated a large spectrum of Ashwagandha effects in mildly anxious, healthy subjects (HS) for 60 days in total. The results highlight a significant dose-dependent effect of this extract with a 41% reduction in anxiety levels and a general improvement in stress and depressive symptoms. The therapeutic mechanism was contextualized with also a significant reduction in cortisol levels and a promotion of dehydroisoandrosterone-sulphate levels, an index of the functioning of the suprarenal glands [84].

### 2.9. Ginseng Extract

Ginseng, a popular herbal remedy derived from the roots of the *Panax* species, has gained attention for its potential neuroprotective and immunomodulatory properties [85]. Ginseng contains bioactive compounds, such as ginsenosides, polysaccharides, and flavonoids, which exert diverse pharmacological effects [86]. In the context of MS, ginseng may modulate immune responses, reduce neuroinflammation, and promote neuroprotection through downregulating the p38 mitogen-activated protein kinase and nuclear factor-κB signaling pathways [87]. Ginsenosides, the primary active components of ginseng, have been shown to inhibit pro-inflammatory cytokine production, regulate immune cell function, and enhance antioxidant defenses [88]. Ginseng supplementation has been shown to attenuate clinical symptoms, reduce inflammatory lesions in the CNS, and preserve neuronal integrity in mice with EAE, immunosuppressing Tregs [89]. Additionally, ginseng extracts have demonstrated neuroprotective effects by promoting remyelination, enhancing synaptic plasticity, and improving cognitive function [90].

### 2.10. Ginkgo biloba Extract

Among the herbal extracts, *Ginkgo biloba*, a remedy obtained from the leaves of the *Ginkgo biloba* tree, displays potential neuroprotective and anti-inflammatory activities due to bioactive compounds, such as flavonoids and terpenoids. The greatest effects, however, seem to depend on acute changes in brain–blood flow, glucose utilization, and increased acetylcholine production [91]. Nevertheless, a randomized controlled pilot study showed that the treatment of patients with MS had very modest effects after treatment with *Ginkgo biloba* extracts [92].

## 3. Diet and MS

### 3.1. Ketogenic or Mediterranean Diet

Focusing on a well-rounded approach, the ketogenic diet (KD) represents a relevant nutritional strategy as an adjuvant in MS treatment (Table 1) [93]. KD is a dietary regimen in which most of the caloric intake and macronutrients derive from lipid-rich foods, the intake of protein is adequate, and carbohydrates are drastically lowered (Table 2). This nutritional protocol aims to induce a state of ketosis, which has been observed to play a positive role in neuroinflammatory pathologies with a reduction in the oxidative damages linked to metabolic stress, an enhancement of signaling cascades related to mitochondrial biogenesis, and a reduction in the release of pro-inflammatory cytokines [93,94]. The standard KD is composed of 70–80% of the daily calorie intake from lipids, less than 50 g/day of carbohydrates, and 1–1.5 g of protein/day, and it determines beneficial effects in patients with MS; however, there are certain risks to be considered, including metabolic acidosis, nephrolithiasis, the onset of dyslipidemia, nutritional deficiencies, and the appearance of changes in bowel habits.

The KD goal is the induction of ketosis, a metabolic state characterized by the high production of ketone bodies used as a source of energy for the body as a substitute for glucose. Ketones can cross the BBB and provide a readily available energy substrate for neurons, particularly when the glucose metabolism is impaired. The ketones deriving from KD are more efficiently metabolized than glucose, producing less Reactive Oxygen Species (ROS) and oxidative stress, and consequently improving the mitochondrial function and enhancing the energy metabolism. This reduction in oxidative stress may protect the neurons from damage and contribute to the KD’s neuroprotective effects [93,94,95]. In this context, the “Phase II study of KDs in relapsing MS: safety, tolerability and potential clinical benefits” by J. Nicholas Brenton et al. [96], published in 2022 in the Journal of Neurology, Neurosurgery & Psychiatry, addressed the KD impact on patients with RRMS, focusing on physiological and immune function throughout the analysis of the clinical and laboratory parameters. This work presents a follow-up of a pilot study conducted on a cohort of 20 patients with RRMS, which managed to test safety, tolerability, and adherence to the modified Atkins KD (MAKD), defined as a ketogenic nutritional protocol consisting of the intake of 65% of the total daily calories from lipids, <20 g per day of carbohydrate, and the remaining percentage of the calorie intake as protein [97,98]. A total of 65 patients aged 12–55 years with a diagnosis of RRMS and disease-modifying therapy were selected to be part of a 6-month prospective study with the intention of treatment with MAKD. Adherence was monitored using daily urine tests to assess the ketone bodies’ presence. Among the parameters evaluated were physical distress, depression, quality of life, fasting adipokines, and clinical biomarkers specific to the disease. Furthermore, patients were provided with the EDSS to examine the elements of disabilities. The exclusion criteria were a progressive MS diagnosis, ongoing gestation, a state of underweight, and the adherence to a ketogenic protocol in the 6 months prior. Patients were encouraged not to exceed 20 g/day of carbohydrates while increasing the consumption of healthy fats.

**Table 1:** Ketogenic dietary intervention studies for MS.

Study Type	Population	Results	Reference
Prospective case-control study	25 patients with MS	6 months of KD intervention enhanced microbial mass and composition.	[99]
Prospective, mixed, and quasi-experimental pilot study	27 patients with MS	12 weeks of KD intervention following the Mediterranean diet food pattern determined enhanced satiation and improved body composition.	[96]
Single-arm, open-label study	20 patients with RRMS	Positive results regarding safety, tolerability, and adherence to MAKD	[97]
6-month prospective intervention	65 patients with RRMS	6 months of intervention with KD determined improvements in anthropometric measures, clinical outcomes, and laboratory biomarkers.	[98]
3-month follow-up of a 6-month prospective intervention	65 patients with RRMS	The KD intervention resulted in improvements in energy levels, cognitive functions, and mood of the patients involved. Adherence of patients during the follow-up varied among participants.	[100]
Randomized Controlled Trial	60 patients with MS	After 6 months of adaptive KD, results highlight reduced serum neurofilament light chain protein (a biomarker of neuroaxonal damage)	[101]

**Table 2:** Formulations of common KDs.

Diet	Percent Total Daily Energy Intake		
	Fat (%)	Carbohydrate (%)	Proteins (%)
Classic KD (4:1 kd)	90	2	8
3:1 KD	87	4	9
2:1 KD	82	8	10
1:1 KD	70	10	20

The daily integration of multivitamins and minerals was also recommended and patients with low calcemia levels were instructed to assume 500–600 mg/day of calcium. Overall, 91% of patients at the baseline were already under vitamin D supplementation, which remained unchanged during the study. The outcomes were measured at baseline, 3 months, and 6 months. The clinical biomarkers were obtained in a fasting state and included the full metabolic panel, insulin, glycated hemoglobin, the lipidic profile, carnitine, vitamin D, adipokines, leptin, and adiponectin. By the end of the study, the most common side effects reported were mainly related to the gastrointestinal system, with a 43% prevalence of constipation, 18% diarrhea, and 9% nausea. Increases in body weight, fatigue, worsening of anxiety, depression, or onset of acne were reported in approximately 5% of patients. Importantly, 13 out of the 48 women evaluated at the end of the study reported irregularities in the frequencies or entity of menstruation. No patients exhibited relapse episodes. The resulting outcomes of the clinical trial highlighted a remarkable decrease in BMI, waist circumference, and fat mass, and a slight reduction in the resting metabolic state, with a 4% increase in lean mass. All the sub-scores about quality of life, fatigue, depression, anxiety, walking speed, and resistance to the 6-min walking test reported amelioration at the end of the study. Specifically, sub-scores with the most prominent improvements were related to physical health, energy, cognitive function, and overall well-being. At the end of the 6-month intervention period, a reduction was also observed in the neurological disability score, with no significant changes in the timed 25-foot walk, the Paced Auditory Serial Addition test, or the Symbol Digit Modalities test. In the context of the blood parameters, at the end of the first 3 months of the study, a carnitine deficit was present in 24% of patients, while the vitamin D levels went positively up. The reasons behind the improvements in vitamin D bioavailability were suggested to be a higher intake through the diet and reduced storage linked to a lower BMI and fat mass.

Notably, ameliorating effects were also present in the glycemic and lipid–blood panel, with a notable reduction in insulin and fasting glycated hemoglobin, and a rise in both LDL and HDL. Among the biomarkers of interest, a reduction in the level of leptin, a pro-inflammatory cytokine, was detected at 3 and 6 months during the dietetic intervention, with an improvement also in the level of adiponectin. In both cases, the variation in these two adipokines was observed to be correlated with the reduction in BMI [98]. BMI and obesity are particularly important parameters to modulate in patients with MS. Studies on a large cohort of patients with MS in Germany revealed a significant correlation between obesity and the worsening of clinical outcomes in patients with MS. Obese individuals featured more extensive brain lesions than their non-obese counterparts, higher rates of relapse episodes, and higher disability scores [102]. This study represents an important reference in the context of the dietary treatment of MS. An 83% adherence was observed with only mild side effects, which were still easily manageable through nutritional profile adjustments. The only relevant downside encountered was the frequent menstrual irregularities because of the hormonal impact of this diet [98].

The Mediterranean diet (MD) is recognized as one of the healthiest dietary lifestyles and the correlation between this nutritional pattern and the lower incidence of several diseases, including neurodegenerative ones, had been widely studied [103,104,105,106]. Traditionally, this dietary pattern features the daily consumption of whole grains, extra virgin olive oil, fruits, and vegetables, and a high-to-moderate weekly intake of legumes, fish, and white meat (Table 3) [107].

Another clinical trial was conducted focusing on the impact of coconut oil and EGCG supplementation within a dietary protocol that follows the MD’s pattern. In this case, patients were instructed to follow a modified version of the MD with a higher intake of lipids. By extension, the diet followed by both groups included the three macronutrients distributed as follows: 20% proteins, 40% carbohydrates, and 40% Mediterranean lipids. The parameters of interest were IL-6 levels, anxiety, and disability related to MS. IL-6, a multifunctional cytokine protein with both pro- and anti-inflammatory properties, acts as a chemical messenger regulating the immune response and can be considered a marker of the activation of the immune system [109]. In the context of MS, IL-6 levels are markedly high, and it has been shown that in affected patients, T cells have a higher number of receptors for this protein [110]. In addition, IL-6 levels are also correlated with the adiposity levels and the degree of anxiety of the subject. IL-6 can influence adiposity through various mechanisms. It has been shown to regulate adipocyte differentiation and lipid metabolism, and it can modulate insulin sensitivity and glucose homeostasis. Additionally, IL-6 has been implicated in the regulation of appetite and energy balance: elevated IL-6 production may contribute to low-grade systemic inflammation associated with obesity, a state often referred to as meta-inflammation [111]. Interestingly, a correlation was found between anxiety and IL-6 levels and how the psycho-physical process induces the recruitment of monocytes related to IL-6 levels. The relationship between inflammation, anxiety, and depression has also been investigated in the UK biobank and the Netherlands Study of Depression and Anxiety cohort. The data suggest that inflammation may be specifically associated with certain symptoms of depression and anxiety. The altered biomarkers featured elevated levels of C-reactive protein, numerous interleukins, and TNF-alpha [112]. Nevertheless, anxiety levels significantly affected the degree of fatigue and functional disability in patients with MS.

### 3.2. D-Galactose

Untargeted metabolomics studies can detect the most prevalent metabolites in patients’ serum, which can be contextualized to better comprehend the disease biomarkers and progression [113]. In this context, an untargeted metabolomic study was conducted to highlight the differences in the serum metabolite levels of HS and patients with RRMS and PPMS. Two-dimensional gas chromatography was used for the comprehensive metabolomics analysis and the resulting metabolite profiles showed clear differences between the different groups. The pathways related to amino acids and sugar metabolism have been found to be affected, by extension, patients with RRMS exhibited lower levels of Gal, implied in the synthesis of myelin, than the HS group. In addition, the paper reports the pivotal role of Gal and its metabolism in MS and other neurological dysfunctions, such as Alzheimer’s disease, exerting beneficial effects by promoting the remyelination processes and favoring cognitive functions. Considering this data and the positive impact of Gal in patients with MS cited in the first part of this review, the role of this monosaccharide and its possible integration into the diet might be important factors to better study. Differently, higher levels of alpha-D-glucose were detected in patients with RRMS compared with the HS group, suggesting a condition of insulin resistance [114]. The involvement of Hexose-6-phosphate dehydrogenase (H6PD) as the initial component of a pentose phosphate pathway located inside the myelin that generates NADPH, to scavenge ROS, has been proposed [115]. A genome-wide association study provided evidence for the H6PD gene as a new candidate gene for MS, as some variants attenuating or abolishing H6PD activity were positively correlated with MS [116]. Insulin receptors are widely represented in the areas of the brain related to memory and cognition, such as the hippocampus. Considering that glucose is the elective source of energy for neurons, reduced responsiveness at this level might lead to compromised neuronal function and survival. Moreover, insulin resistance has been correlated with neuroinflammation by triggering microglia activation, which, once activated, is responsible for the release of pro-inflammatory cytokines and ROS, leading newly to neuronal damage [117].

### 3.3. β-Hydroxybutyrate

To counteract the general inflammation state in MS, ketone bodies obtained through the beta-oxidation of coconut oil have been related to improvements in the inflammatory markers, the lipidic panel, the level of glycated hemoglobin, and the reactive protein C concentration. In patients with MS, the ketonic body *β*-hydroxybutyrate activates the hydroxycarboxylic acid receptor 2 receptors expressed at the level of the neuroinflammatory cells, reducing neuroinflammation and, consequently, carrying out a neuroprotective effect.

### 3.4. Other Dietary Supplements

Several molecules discussed in Section 2 can also be considered as dietary supplements. However, other molecules, such as vitamins or essential fatty acids, that play a fundamental role in modulating inflammation and regulating oxidative stress in MS also compose the dietary supplements proposed for MS. Among those most widely used are [118]:

1. Omega-*3* fatty acids, such as eicosapentaenoic acid and docosahexaenoic acid, exert anti-inflammatory and neuroprotective effects. It was demonstrated that supplementation with omega-3 fatty acids has been associated with reduced relapse rates, improved cognitive function, and enhanced quality of life in patients with MS.
2. Vitamins A, C, and E display a positive effect on MS progression as antioxidant molecules, scavenging free radicals and reducing the oxidative stress production. However, they also mitigate inflammation, helping to preserve neuronal integrity and function in MS.
3. A separate mention is deserved for vitamin D, the deficiency of which is associated with the onset and progression of MS, as this molecule is involved in the modulation of the immune response. Supplementation with vitamin D has shown promising results in reducing disease activity and progression.
4. Probiotics. Since emerging evidence suggests a link between gut microbiota dysbiosis and MS pathogenesis, probiotic supplementation could act in immune response modulation.

## 4. Conclusions

Although MS is considered an autoimmune disease, patients display defects in energy metabolism and redox balance, which result in increased oxidative stress and the associated inflammatory responses. These molecular mechanisms could explain why, despite advances in pharmacological therapies, many individuals with MS seek complementary and alternative treatments to alleviate their symptoms and improve their quality of life. Therefore, in recent years, there has been an effort to supplement traditional pharmacological treatments with natural bioactive compounds with antioxidant and anti-inflammatory effects, either through dietary supplementation or changes in the diet, without aiming to replace the official therapies. Accumulating evidence indicates that the natural compounds for MS present few side effects. A recent review addressed this issue, summarizing 80 studies on natural compounds active on MS from three aspects: immune regulation, oxidative stress suppression, and myelin protection and regeneration in MS. There is preclinical evidence that natural compounds may attenuate MS progression by suppressing immune attacks and promoting myelin repair [119]. However, much remains to be clarified, as while the effect of the antioxidant and anti-inflammatory molecules appears promising in in vitro studies in cellular and animal models, the results of human clinical trials are much less clear and require further studies to verify the therapeutic efficacy and safety. On the other hand, from a future perspective, a therapy based not only on the use of drugs but also on a healthy lifestyle and proper nutrient supplementation could represent the path to making MS treatment customizable.

## Figures and Tables

**Table 3:** The MD composition according to Bach-Faig A., et al. [108].

Foods	Frequency
Olive oil	Every meal
Vegetables	>2 serves every meal (one raw)
Legumes	≥2 serves weekly
Nuts	1–2 serves daily
Fruits	1–2 serves every meal
Dairy foods (low fat)	2 serves daily
Red meat	<2 serves/week
Fish/shellfish	≥2 serves weekly
Eggs/poultry	2–4 serves weekly
Cereals (whole grain)	1–2 serves every meal

## Data Availability

Not applicable.

## Abbreviations

MS	Multiple Sclerosis
CNS	Central Nervous System
Relapsing-Remitting Multiple Sclerosis	RRMS
Disease-modifying therapies	DMT
Secondary Progressive Multiple Sclerosis	SPMS
Primary Progressive Multiple Sclerosis	PPMS
Nervous System	NS
Myelin Basic Protein	MBP
D-Galactose	Gal
Coenzyme Q10	CoQ10
Matrix Metallo Proteases	MMPs
Blood-Brain Barrier	BBB
Tumor Necrosis Factor Alfa	TNF-α
Interleukin-6	IL-6
Interleukin-4	IL-4
Superoxide Dismutase	SOD
Glutathione Peroxidase	GPx
Expanded Disability Status Scale	EDSS
Cuprizone	CPZ
Resveratrol	RV
Nuclear Factor Erythroid 2-Related Factor 2	Nrf-2
Interleukin-1β	IL-1β
Regulatory T cells	Treg
Experimental Autoimmune Encephalomyelitis	EAE
Epigallocatechin gallate	ECGC
Body Mass Index	BMI
Citicoline	DCI
Phosphatidylcholine	PC
Oligodendrocyte Precursor Cells	OPCs
Boswellic Acid	BA
Heme-oxygenase 1	HO-1
Ketogenic Diet	KD
Mediterranean Diet	MD
Reactive Oxygen Species	ROS
Modified Atkins Ketogenic Diet	MAKD
Healthy Subjects	HS
Hexose-6-phosphate dehydrogenase	H6PD
